# Machine learning-based approaches for ubiquitination site prediction in human proteins

**DOI:** 10.1186/s12859-023-05581-w

**Published:** 2023-11-28

**Authors:** Mahdi Pourmirzaei, Shahin Ramazi, Farzaneh Esmaili, Seyedehsamaneh Shojaeilangari, Abdollah Allahvardi

**Affiliations:** 1 Department of Information Technology, Tarbiat Modares University, 14115-111 Tehran, Iran; 2 Department of Biophysics, Faculty of Biological Sciences, Tarbiat Modares University, 14115-111 Tehran, Iran; 3 Biomedical Engineering Group, Department of Electrical and Information Technology, Iranian Research Organization for Science and Technology (IROST), 33535111 Tehran, Iran

**Keywords:** Ubiquitination, Deep learning, Machine learning, Post-translational modification

## Abstract

Protein ubiquitination is a critical post-translational modification (PTMs) involved in numerous cellular processes. Identifying ubiquitination sites (Ubi-sites) on proteins offers valuable insights into their function and regulatory mechanisms. Due to the cost- and time-consuming nature of traditional approaches for Ubi-site detection, there has been a growing interest in leveraging artificial intelligence for computer-aided Ubi-site prediction. In this study, we collected experimentally verified Ubi-sites of human proteins from the dbPTM database, then conducted comprehensive state-of-the art computational methods along with standard evaluation metrics and a proper validation strategy for Ubi-site prediction. We presented the effectiveness of our framework by comparing ten machine learning (ML) based approaches in three different categories: feature-based conventional ML methods, end-to-end sequence-based deep learning (DL) techniques, and hybrid feature-based DL models. Our results revealed that DL approaches outperformed the classical ML methods, achieving a 0.902 F1-score, 0.8198 accuracy, 0.8786 precision, and 0.9147 recall as the best performance for a DL model using both raw amino acid sequences and hand-crafted features. Interestingly, our experimental results disclosed that the performance of DL methods had a positive correlation with the length of amino acid fragments, suggesting that utilizing the entire sequence can lead to more accurate predictions in future research endeavors. Additionally, we developed a meticulously curated benchmark for Ubi-site prediction in human proteins. This benchmark serves as a valuable resource for future studies, enabling fair and accurate comparisons between different methods. Overall, our work highlights the potential of ML, particularly DL techniques, in predicting Ubi-sites and furthering our knowledge of protein regulation through ubiquitination in cells.

**Supplementary Information:**

The online version contains supplementary material available at 10.1186/s12859-023-05581-w.

## Introduction

In all eukaryotic cells, post-translational modification (PTM) of proteins takes place after the ribosome has translated the mRNA into the protein. PTM can change the structure, electrophilicity, and interactions between the proteins [1]. Often, PTMs on proteins are considered reversible or irreversible processes in cells. Nevertheless, reversible PTMs play vital roles in extending the functional diversity of proteins and significantly affect the regulation of protein functions in eukaryotic organisms. PTMs have emerged as crucial molecular regulatory mechanisms that are utilized to regulate diverse cellular processes [2]. PTMs can alter the properties of a protein or lipoprotein by proteolytic cleavages or by adding different functional groups to proteins during or after synthesis, such as phosphoryl, glycosyl, acetyl, and methyl groups to one or more amino acids in sequences [2]. The UniProt database currently lists over 600 known PTMs [3]. While PTM modifications have been identified in the past few decades, few have been fully characterized functionally [4]. PTMs can be found in various parts of the cell and participate in a wide range of biological processes, including DNA repair, gene expression, regulation and activation, cell cycle control, and signal transduction [2]. PTMs play significant roles in the structure and function of proteins and, as a result, regulate diverse molecular processes such as folding, localization, interactions, and degradation. [5]. Disorders in PTMs-dependent protein and lipoprotein regulation have been linked to the onset and progression of various diseases, including cancer, cardiovascular, renal, and neurodegenerative diseases [3, 6].

**Fig. 1:**
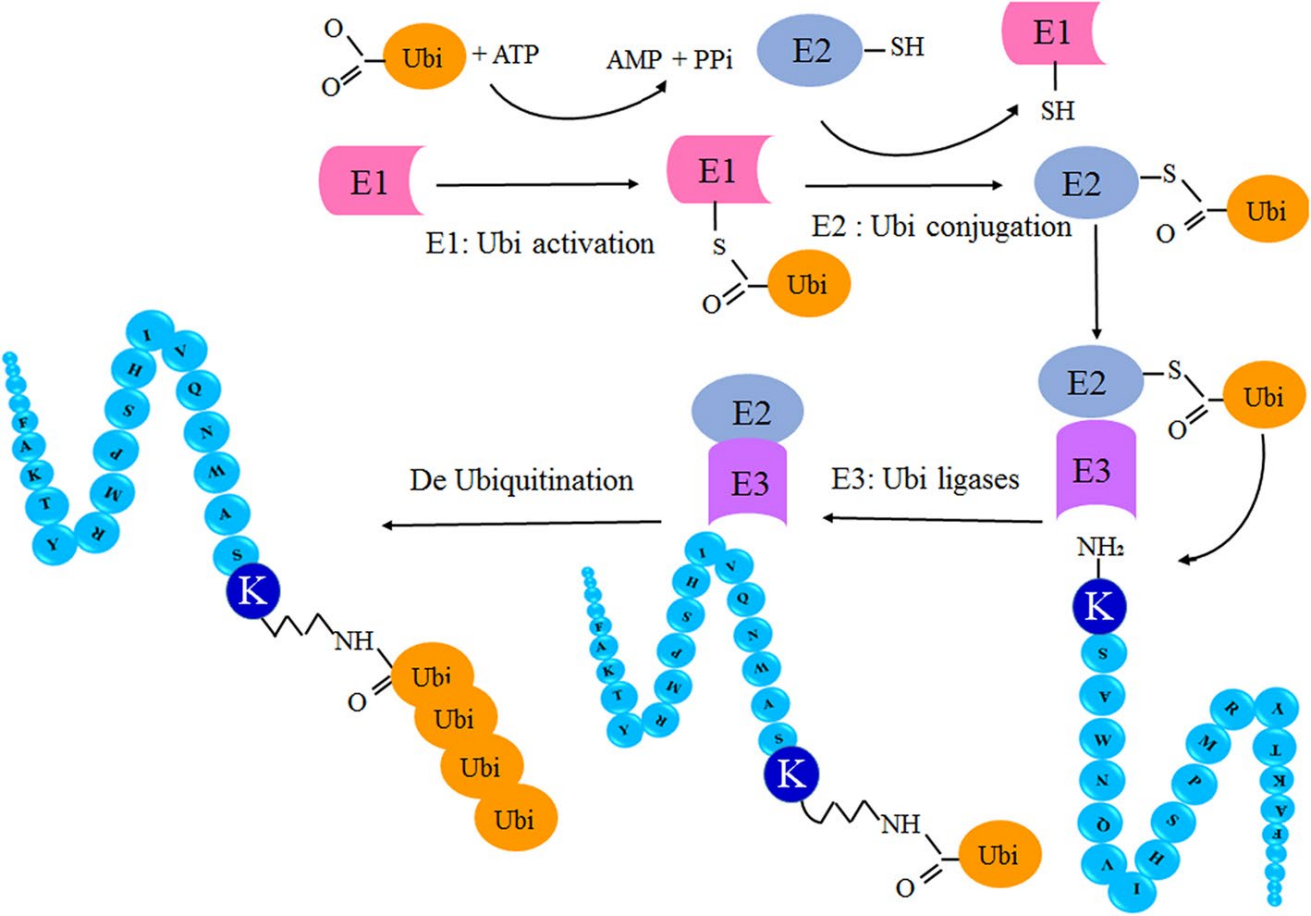
The cycle of reversible ubiquitination process

Ubiquitination was first reported by Gideon Goldstein as a polypeptide named ubiquitous immunopoietic polypeptide (UBIP), which was isolated from bovine or human thymus in 1975 [7]. Ubiquitination is a reversible PTM on proteins. Ubiquitin, a 76-amino acid protein, is covalently attached through an isopeptide bond between its C-terminal glycine and Nε lysine (K). This modification is highly conserved throughout eukaryotic organisms [8, 9]. Furthermore, ubiquitination of lysine in histone proteins can modify chromatin structure [10]. Previous studies have reported that ubiquitination modification of H2A and H2B histone proteins plays a key role in regulating chromatin dynamics and gene transcription [9, 11]. This reversible reaction can affect protein concentration, folding, localization, and aggregation [12]. Ubiquitination can occur as monoubiquitin as well as polyubiquitin on lysine residues in the target protein, which can induce complex topologies. However, monoubiquitination modifications occur more frequently and play important roles in various cellular processes [13]. Lysine-ubiquitinated proteins may be modified by ubiquitin-like molecules, such as the small ubiquitin-like modifier (SUMO) or neuronal precursor cell-expressed developmentally down-regulated protein 8 (NEDD8). Furthermore, ubiquitinated proteins have been shown to be phosphorylated on serine, threonine, or tyrosine residues or acetylated on lysine residues, and each modification can significantly change the signaling pathway's outcome [13]. Three enzymes, including an activating enzyme (E1), a conjugating enzyme (E2), and an ubiquitin ligase (E3), are involved in the ubiquitination process (Fig. 1). The ubiquitin can be separated from the protein through deubiquitinating enzymes (DUBs) [14]. Ubiquitination plays essential roles in nearly all aspects of eukaryotic biology and in many cellular processes such as proteasome and lysosome degradation, gene transcription, DNA repair and replication, intracellular trafficking, stress response, cell-cycle regulation, endocytosis signaling, transcriptional regulation, virus budding, and subcellular localization [8, 9, 14, 15]. The alteration of the ubiquitination system is closely related to cellular transformation, the immune response, and the inflammatory response. Therefore, the regulatory function of the ubiquitin–proteasome system plays a vital role in cellular homeostasis [15]. Disruption of this type of PTM can lead to cancer, autoimmunity, inflammatory disorders, diabetes, and neurodegenerative diseases [16, 17].

To identify ubiquitination and various kinds of PTM, three methods are used: mass spectrometry (MS), immunoprecipitation (IP), and proximity ligation assay (PLA) [18]. However, the MS method is considered a superior method for detecting, mapping, and quantifying ubiquitination in human proteins [3]. However, due to the costly and time-consuming nature of these traditional approaches for detecting different types of PTMs and Ubi-sites [19], there has been a growing interest in leveraging artificial intelligence for computer-aided Ubi-site prediction.

Given the importance of the topic, it is surprising that little research effort has been focused on Ubi-site prediction in human proteins with no agreement on their methodologies or evaluations, and therefore, there are no suitable tools for automating the prediction process.

**Fig. 2:**
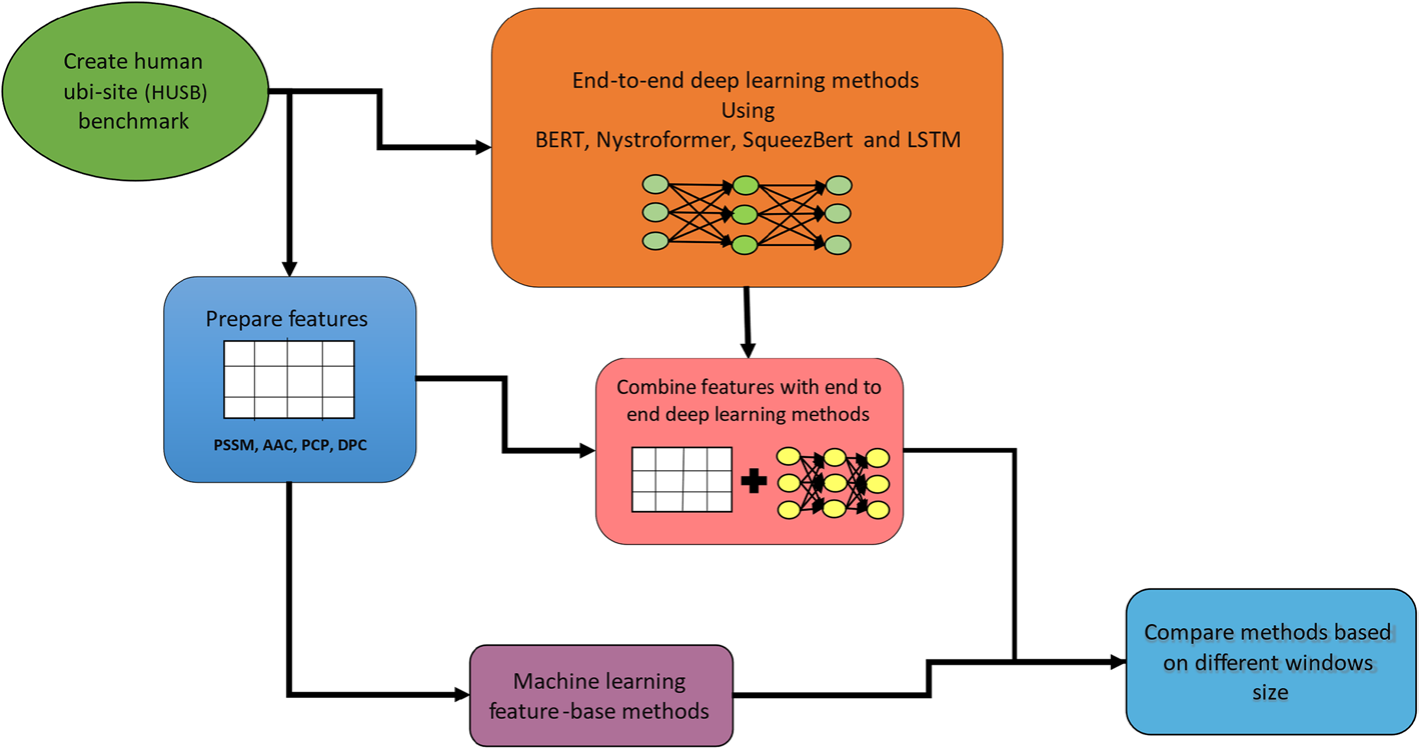
The flow diagram of this work

The main contribution of this article is to develop a benchmark for a fair comparison among different ML-based Ubi-site prediction models for researchers. Extensive experiments were conducted to investigate the effects of both conventional machine learning (ML) and end-to-end deep learning (DL) models on Ubi-site prediction. Additionally, a hybrid approach was introduced that combines hand-crafted features with raw protein sequences as input for a deep neural network (DNN) architecture. To ease the predicted model comparison, we designed a benchmark with open-access datasets (collected from the dbPTM 2019 and dbPTM 2022 databases), standard evaluation metrics, and the proper validation strategy to avoid information leakage. The proposed benchmark is available on the GitHub repository (https://github.com/mahdip72/ubi) for other researchers to compare their work in a standard setting. We presented the effectiveness of our framework by comparing ten ML-based approaches in three different categories: feature-based conventional ML methods, end-to-end sequence-based DL methods, and hybrid feature-based DL methods. Overall, these contributions provide new insights into the use of ML approaches for predicting Ubi-sites and further advance our understanding of protein regulation through ubiquitination. The schematic flow of this work is presented in Fig. 2.

### Related works

Several Ubi-sites have been identified through the advancement of the high-throughput MS method. However, large-scale ubiquitination detection is costly, labor-intensive, and challenging. Therefore, a lot of attention has been paid to computational methods, such as traditional ML and DL, for predicting Ubi-sites in recent years. By analyzing existing experimental data via available PTM databases and identifying relevant features, ML algorithms can significantly reduce costs and labor in detecting potential Ubi-sites in human proteins. Currently, there is no useful computational tool available to predict Ubi-sites for human data, despite the crucial need for a more cost- and time-effective alternative to experimental approaches. Nevertheless, for the prediction of ubi-sites in protein sequences across various species, ML approaches use different algorithms, such as random forest (RF) [20], extreme gradient boosting (XGB) [21], support vector machine (SVM) [22], K-nearest neighbor (KNN) [23], and others that we will briefly review. Tong and Ho [24] examined different features with various classifiers like SVM, Naïve Bayes, and KNN and concluded that the physicochemical properties (PCPs) with the SVM classifier obtained the best results. Radivojac et al. [25] developed the UbPred tool using a RF classifier to predict ubi-sites in *Saccharomyces cerevisiae*. In this study, for training the model, 157 Ub-sites were extracted from a database of ubiquitinated proteins via sequence and structural-based features. UbPred achieved the best results with an accuracy of 72%, and the area under the ROC curve at 80%. Cia et al. [26] employed multiple ML classifiers to identify ubi-sites based on the PCPs of amino acids in protein sequences. The models' resilience and prediction accuracy were evaluated using fivefold cross-validation, and the results showed that EBMC, SVM, and LR algorithms have performed better than other methods [26] and EBMC has AUCs greater than or equal to 0.6. Another study [27] showed that the combination of amino acid composition (AAC) and the composition of k-spaced amino acid pairs (CKSAAP) features as input to the SVM classifier got 81.56% AUC in fivefold cross-validation and 86% AUC via an independent test for *Arabidopsis thaliana* Ubi-sites. In recent years, DL algorithms, as more advanced techniques, have become increasingly popular for predicting Ubi-sites due to their success in handling large-scale data. For instance, DeepUni [14] proposed an algorithm based on a convolutional neural network (CNN) for predicting Ubi-sites using four different sequence-base features and PCPs and achieved a 0.99 AUC. Liu et al. [28] developed a novel transfer DL method called DeepTL-Ubi for predicting Ubi-sites across multiple species. Their proposed model was trained and tested on data from several sources and demonstrated improved predictive performance for species with small sample sizes compared to other tools. Wang et al. [29] employed an improved word embedding scheme based on a transfer learning strategy, which was combined with a multilayer CNN to identify Ubi-sites in plant proteins. The proposed classifier achieved an AUC of 0.82, outperforming other ML-based methods. He et al. [30] utilized a multimodal deep architecture for identifying Ubi-sites based on three methods: raw protein sequence fragments, PCPs, and sequence profiles. Finally, the generative deep representations corresponding to these three modalities were merged to build the final model, which achieved a performance of 66.43% AUC. Using various sequence-base features, including binary encoding (BE), pseudo-amino acid composition (PseAAC), the composition of CKSAAP, and position-specific propensity matrices (PSPM), Cui et al. [31] developed an SVM-based algorithm on three datasets (Set1, Set2, and Set3). Next, LASSO was used to remove redundant feature information and select the optimal feature subset. The UbiSitePred [31] model demonstrated better prediction performance compared to other evaluated methods through fivefold cross-validation. The model achieved AUC values of 0.9998, 0.8887, and 0.8481 and accuracy rates of 98.33%, 81.12%, and 76.90% for Set1, Set2, and Set3, respectively. In addition to the mentioned approaches, similar research has also employed DL techniques for protein classification. For instance, in reference [32], a deep RF algorithm was utilized to achieve 96% accuracy in classifying golgi proteins. Moreover, in the paper [33], a classification model was developed to classify phage virion proteins. This model utilized the UniRep feature for protein sequence quantification and employed the LightGBM algorithm for evaluation.

Although a few studies have been conducted on Ubi-site prediction in human proteins [34, 35], there is no agreement on their methodologies or evaluations. Therefore, there are no suitable tools for automating the prediction Ubi-sites. In this study, a significant amount of ubiquitination data was extracted from a publicly accessible database and processed to create a well-defined benchmark. These resources play a pivotal role in uncovering intricate patterns within the landscape of protein ubiquitination and lay the foundation for the development of innovative models aimed at predicting Ubi-sites. By employing advanced computational methods and algorithmic approaches, researchers can delve into the complex interplay between ubiquitin molecules and their target proteins. Understanding the importance of predicting Ubi-sites in the human species holds the potential to unravel key regulatory mechanisms that influence cellular processes ranging from protein turnover to signal transduction. The utilization of specific methodologies and algorithms in this context enables scientists to unravel the dynamic orchestration of PTMs, shedding light on how ubiquitination impacts health, disease, and the overall functioning of biological systems.

## Methodology

### Machine learning methods

ML is a rapidly growing field that has gained increasing attention from both academic and industrial research. Indeed, ML encompasses the employment of statistical techniques and computational methodologies to autonomously discern patterns within data, subsequently refining a model or performance criterion through this acquired knowledge [36]. ML algorithms have been widely applied in various domains of biological research, including the prediction of PTMs in proteins. The success of ML in these contexts is partly due to its ability to process large volumes of data and identify complex patterns that may be difficult for humans to discern. In the past decade, the emergence of DL has revolutionized whole fields related to artificial intelligence and led to the development of two main categories of ML techniques: conventional or classical approaches, which typically rely on handcrafted features and feature engineering, and end-to-end DNNs, which are capable of learning and extracting features from raw data (e.g., sequences of amino acids), eliminating the need for manual feature engineering. While both types of methods have their own strengths and limitations, the use of end-to-end DNNs has become increasingly popular in recent years due to their ability to handle large and complex tasks and their ability to learn directly from the data.

#### Conventional methods

In our study, XGBoost [21], SVM [22, 37], KNN [38], RF [20], and ANN [39] have been utilized. In the following, a brief explanation of the main concept of each method has been provided.

*XGBoost* It is an ensemble learning algorithm based on gradient boosting and an optimization model that combines a linear model with a boosting tree model. The algorithm learns a series of decision trees to classify the labeled training data. Each decision comprises a series of rules that semi-optimally split the training data. Successive trees that “correct” the errors in the initial tree are then learned to improve the classification of positive and negative training examples.

*SVM* It is a type of supervised ML algorithm that can be used for classification or regression tasks [40]. It works by finding the hyperplane in a high-dimensional space that maximally separates the different classes. The distance between the hyperplane and the nearest data points is known as the margin, and the goal of SVM is to maximize this margin. In the case of non-linearly separable data, SVMs can still be used by transforming the data into a higher-dimensional space using a kernel function, which allows the data to be linearly separable in the higher-dimensional space. SVMs are particularly useful for problems with high-dimensional data and can be effective in cases where there are a limited number of training examples. They are also resistant to overfitting, meaning that they generally perform well on unseen data. SVMs have been widely used in a variety of applications, including text classification, image classification, and bioinformatics [37].

*KNN* This algorithm is a simple, non-parametric method used for classification and regression. It works by identifying the k number of training examples that are closest in distance to the new input point and assigning the label of the majority of those points to the new input. The distance can be calculated using any distance metric, such as the Euclidean or Manhattan distance. KNN is considered a lazy learning algorithm because it does not build a model but instead simply stores the training data and makes predictions based on the similarity of the new input to the stored training examples. It is often used when the relationship between the features and the output is not well understood and can be effective for tasks such as image classification and text classification. However, KNN can be computationally expensive and may not scale well to large datasets [23, 38].

*RF* It is an ensemble learning method that combines the predictions of multiple decision trees to make a more accurate and stable prediction. It works by training a large number of decision trees on randomly selected subsets of the training data and using the average or majority vote of the individual trees to make a final prediction. Each tree in the forest is trained using a random subset of the features, which helps to reduce overfitting and improve the generalizability of the model. RF is widely used for classification and regression tasks and is known for its good performance, robustness, and ability to handle large and complex datasets. It is also resistant to overfitting and can be used for feature selection, as the importance of each feature can be determined by how much it contributes to the decision made by the tree [20, 41].

*ANN* Inspired by the architecture and operation of the human brain, artificial neural networks (ANNs) are computer models made up of interconnected “neurons” that process and transfer data. ANNs consist of multiple layers, with the input layer receiving the input data, the output layer producing the output, and one or more hidden layers in between. The weights of the connections between the neurons are adjusted during the training process, allowing the network to learn and adapt to new data. DNNs are able to learn and generalize patterns in data and are commonly used for tasks such as classification, prediction, and clustering [39].

#### Deep learning methods

*DL* Based methods rely on raw data to process, meaning they extract features within their layers in an end-to-end fashion. In our study, we examine the effect of DNNs architectures on Ubi-site prediction. Two types of architecture were considered for sequence processing: recurrent neural networks (RNN) and transformers. Both of these architecture types are widely recognized and have a proven track record of success in sequence processing tasks. For the first type, long short-term memory (LSTM) was used, and for the second one, a list of transformers, including the bidirectional encoder representation transformer (BERT), Nystromformer, and SqueezeBERT, was considered. The details of each architecture are described below:

*LSTM* It is a type of RNN that is particularly effective at modeling long-term dependencies in sequential data. It accomplishes this by using “gates” in its hidden layer, which act as filters to selectively remember or forget certain information based on the input data and previous hidden state. The use of gates in LSTM allows the network to adaptively preserve or discard information as needed, effectively capturing dependencies that span many time steps. LSTM has been applied to a wide range of tasks, including language modeling, machine translation, speech recognition, stock price prediction, language translation, and image caption generation. It is especially useful for tasks where the contextual information of preceding words or sounds plays a crucial role in accurately predicting the subsequent ones [42, 43].

*BERT* It has created a major breakthrough in the field of natural language processing (NLP) by achieving state-of-the-art results-related tasks such as question answering, text generation, sentence classification, etc. [44]. BERT relies on a transformer, which is an attention mechanism that learns contextual relationships between words in a text. A basic transformer consists of an encoder for reading the text input and a decoder for producing a prediction for the task at hand. Since BERT’s goal is to generate a language representation model, it only needs the encoder part. The input to the encoder for BERT is a sequence of tokens, which are first converted into vectors and then processed in the neural network.

*Nystromformer* It replaces the self-attention mechanism in BERT-small and BERT-base using the proposed Nyström approximation [45]. This reduces the self-attention complexity to O (n)^1^ and allows the transformer to support longer sequences.

*SqueezeBERT* It is based on the procedures acquired from SqueezNAS, a neural architecture search (NAS) model [46]. The key difference between the BERT architecture and the SqueezeBERT architecture is that the latter uses grouped convolutions instead of fully connected layers, allowing SqueezeBERT to be 4.3× faster than BERT.

### Features

In order to process protein sequences for later classification tasks, it is necessary to encode the input sequences and convert them into numerical feature vectors. Previous research has demonstrated the effectiveness of using multiple characteristics from various sources to provide supplementary information from the protein samples [47]. These sources may include information about the 20 amino acid residues present in the sequence. In our study, five different types of features were used to improve the accuracy of our prediction model. These features are briefly described below. It is worth noting that incorporating multiple characteristics from various sources can help capture additional context and nuance in the data, ultimately leading to more accurate prediction results.

*Sequence-based features* To use an end-to-end DL system to predict Ubi-sites, it is necessary to prepare a sequence of amino acids by performing two steps: sequence encoding and converting the encoded sequence to numerical vectors. There are two commonly used techniques for the latter step: one-hot encoding and word embedding [48, 49]. One-hot encoding is an approach for representing categorical inputs, such as amino acid codes, as numerical vectors. This method is frequently employed as a preprocessing step to prepare data for feeding into DL models. However, in the context of Ubi-site prediction, word embedding is often preferred due to its effectiveness and the similarities between PTM prediction and NLP tasks [29].

*Amino acid composition (AAC)* AAC is one of the most important and widely used sequence-based features for classification problems. AAC encodes the frequency of each amino acid in a peptide sequence of a given length [47]. The AAC is calculated based on Eq. 1 where *ci* represents amino acid *i* in a peptide sequence.

1 $$AAC = \sum {Ci/length} (seq)\;i = 1, \ldots ,20$$

*Dipeptide composition (DPC)* DPC is used to extract the attributes of amino acid compositions, like other composition algorithms [50]. The equation calculates the percentage of the double composition of each amino acid in the sequence, divided by the number of all possible dipeptides generated by 20 amino acids (20 * 20 = 400), which is represented as Eq. 2:

2 $$DPC = \frac{number \;of\; amino\; acid\; product\; occurrence \;in\; sequence}{{total\; dipeptide \;composition \left( {400} \right)}} \times 100.$$

*Physicochemical properties (PCP) *In the early 1970s, Chou suggested PseAAC as a useful way to encode protein sequences. PseAAC [51] contains information about the sequence order and physiochemistry, incorporating different frequencies of the 20 types of amino acids. In past studies, the physicochemical and biological properties of amino acids were extracted from the amino acid index database (AAIndex) [52, 53] and used for predicting PTM sites. In this work, 16 different physicochemical and biochemical properties were analyzed to calculate the PseAAC. These properties were extracted from the AAIndex database by Xiang et al. [54].

*Position-specific scoring matrices (PSSM)* PSSM is an evolutionary-based feature that is highly informative and widely used for protein representation in computational biology studies [55]. The PSSM matrix contains the probability scores of each amino acid incidence in a sequence at each position of the alignment with a set of homologous sequences. The dimension of the matrix is based on sequence length and is defined as L*20. Each row represents an amino acid in sequence, and the columns show 20 amino acids in a protein sequence [56].

### Evaluation metrics

To assess the performance of the Ubi-site prediction models, the following metrics have been reported in this study [57]. It is noted that TP represents true positives (the number of Ubi-sites that have been experimentally validated and accurately predicted by the model), TN stands for true negatives (the number of non-Ubi-sites that have been accurately predicted by the model), FP means false positives (the non-Ubi-sites that have been inaccurately predicted as Ubi-sites), and FN represents false negatives (the experimentally validated Ubi-sites that have been inaccurately predicted as non-Ubi-sites) in the following equations. Moreover, we have reported the Matthews correlation coefficient (MCC) as defined in Eq. 8, to provide a comprehensive assessment of our binary classification model's performance.

3 $$Accuracy = (TP + TN)/(TP + FP + TN + FN) \times 100$$

4 $$Sensitivity(Recall) = TP/(TP + FN)$$

5 $$Specificity = TN/(TN + FP)$$

6 $$Precision = TP/(TP + FP)$$

7 $$F1score = 2TP/(2TP + FP + FN)$$

8 $$MCC = (TP \times TN - FP \times FN)/\sqrt {(TP + FP)(TP + FN)(TN + FP)(TN + FN)}$$

Indeed, for imbalanced classification tasks like Ubi-site prediction, the accuracy rate is an inadequate and unfair metric, despite its popularity in the literature. The F1 score, as defined in Eq. (7), provides a fair measure of the classifier’s performance. Though it is common to report the performance of models using precision, recall, and F1 as described by Eqs. (4) to (7), macro-averaged precision, recall, and F1 were computed to consider both categories equally. The macro-averaging method calculates precision, recall, and F1 for each class first and then averages these statistics for overall categories. This is particularly useful when working with imbalanced datasets, where some classes may be underrepresented. Macro-averaging allows us to see how well each class is being predicted separately rather than considering them as a whole. For example, in a binary classification problem with two classes, class A and class B, if the model has high precision for class A but low precision for class B, the overall precision of the model may be misleadingly high. In this case, calculating macro precision would give a more accurate representation of the model's performance, as it would take into account the precision of both classes. In this work, our main evaluation metric to determine the best approach is the macro-F1 scores as represented by Eq. 9, where ${F1}^{0}$ and ${F1}^{1}$ are F1 scores for non-Ubi-sites and Ubi-sites classes, respectively.

9 $$Macro - F1 = (F1^{0} + F1^{1} )/2$$

## Human Ubi-site benchmark

Prediction of Ubi-sites has been challenging due to the lack of well-defined benchmarks in the research community. While many ML methods have been proposed for this task, it has been difficult to fairly compare their performance due to the use of different test sets and evaluation strategies. This lack of a standardized benchmark makes it difficult to determine the most effective method for Ubi-site prediction and identify areas for improvement. To address this issue, we compiled a comprehensive dataset for human Ubi-site prediction that includes a diverse range of Ubi-sites and a standardized evaluation strategy.

By using such a hold-out^2^ dataset as a benchmark, we aim to compare and improve the performance of ML approaches for Ubi-site prediction. By evaluating the performance of these methods on this separate dataset, we can gain a better understanding of how well they generalize to new data and identify which method may be the most effective for this specific task. In this study, we created a pre-processed, comprehensive, and well-defined ubiquitination benchmark based on human proteins from the dbPTM database [58]. The dbPTM database is the largest available database of experimental results for different types of protein PTMs, including Ubi-sites. In the following sections, we describe the suggested benchmark and explain how to use it in our analysis.

### Data collection and pre-processing

In order to prepare our benchmark, we considered human proteins from two versions of the dbPTM database that included Ubi-sites to create two sets. Initially, all proteins with a length of less than 100 amino acids were removed from the dataset. Since many sequences might be similar to each other and have a negative effect on the classifiers [2, 50], a tool called CD-HIT was used to cluster all the sequences. By setting a threshold value, CD-HIT was used to merge sequence similarities in a large corpus of proteins. The procedure for creating two sets is as follows:

*Set 1* All Ubi-sites in human proteins were gathered from the 2019 release of dbPTM. After performing CD-HIT at 40% on the total collected data (32,407 proteins), there were 5429 proteins remaining [59]. These remaining proteins were considered to be used to build the training and validation sets, which are described below.

*Set 2* In the 2022 release of dbPTM, 7049 new proteins have been collected, and a CD-HIT of 40% has been performed once again to ensure that there are no similar proteins in both protein sets. In the end, 2,348 proteins from the 2022 release were left to build the test set.

**Fig. 3:**
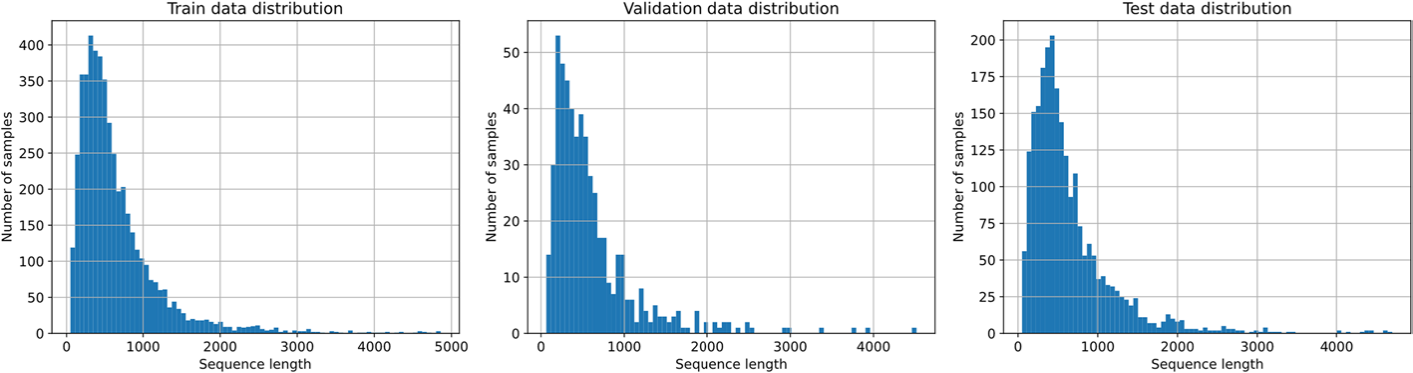
Sequence length distribution: Left: training set. Center: validation set. Right: test set

A systematic ML strategy for the evaluation of the proposed benchmark was adopted, which is similar to the independent-test approach. Considering the two sets described previously, the training, validation, and test sets were prepared as shown in Table 1. Additionally, a graphical representation delineating the distribution of protein lengths across the triad of sets is vividly portrayed in Fig. 3.

**Table 1:** Distribution of positive and negative sites in training, validation, and test sets

Set	# of proteins	# of positive sites	# of negative sites
Training	4886	15,181	180,393
Validation	543	1613	18,989
Test	2348	9713	82,554

*Training set* 90 percent of the proteins are selected randomly from set 1 to create the training set.

*Validation set* The remaining 10 percent of proteins in set 1 are used for the validation set. *Test set* All the proteins in set 2 are considered for the test set.

The development of accurate and reliable predictive models for Ubi- sites is of great importance in the field of bioinformatics. However, the high number of proteins in these datasets presents a challenge when evaluating model performance. Traditional methods such as k-fold cross-validation are not suitable in this case, as they require a relatively small number of samples to be effective.

An independent-test strategy was employed in this study to address this issue. This approach allows us to directly compare the performance of proteins from the 2019 and 2022 releases of dbPTM, which is crucial for assessing the robustness of our models. By minimizing the difference in performance between these releases, we aimed to create predictive models that could generalize well to different protein sets.

Overall, the use of an independent-test strategy is essential for ensuring the reliability and applicability of Ubi-site prediction models. By carefully evaluating their performance on a diverse set of proteins, we can be confident in the accuracy and usefulness of these models for a wide range of research applications.

In this strategy, in contrast to other methods, there were considered to be two unique sets of proteins being investigated. Therefore, the models have less tendency to overfit. Every model introduced in this paper is trained on the training set. During the training procedure, the best model is selected based on the highest macro-F1 value with respect to the validation set. After completing the training and selecting the best model, it is evaluated on the test set, and the desired metrics are reported as the final result.

For a fair and precise comparison between Ubi-site prediction methods, we need uniform, comprehensive, unique, well-defined training, validation, and test sets. Therefore, in this study, by building these three sets, a benchmark named the human Ubi-sites benchmark (HUSB) was created to accomplish a crucial step for future research in this field.

### Craft windows

**Fig. 4:**
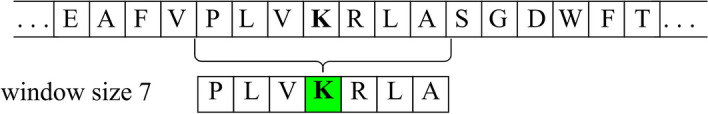
Building fixed-size windows from a protein sequence

Based on different biological features, the total positive or negative samples in each protein sequence were extracted and converted into numerical feature vectors for use in the final classifier. For this encoding, in this step, using a sliding window, all proteins are partitioned into polypeptides with length W, in such a way that the target residue (lysine (K)) is placed at the center of the polypeptides with a fixed-size window (Fig. 4). If the middle amino acid is a Ubi-site, it will be considered a positive label (1); otherwise, it will be negative (0).

During the segmentation of the sequences, at the tail of proteins, the left or right side of some Ubi-sites may be shorter than the fixed window size. To address this, we add padding (unique characters) to the ends of shorter sequences to create windows of fixed length. After creating segmented windows, it was detected that there were two types of duplications present in them. The first type occurs when samples (i.e., both windows and their labels) are the same. In this situation, we kept one sample and removed the rest. The second type occurs when two samples have identical windows but different labels. We removed samples that have negative labels in this situation, as the number of positive sites is generally lower compared to negative sites. This indicates the importance of having a sufficient number of positive labels for effective training. Moreover, positive labels are more reliable because they are based on experimental measurements. In other words, by comparing the 2019 and 2022 versions of dbPTM, we observed that some negative sites have changed to positive sites. It means that since positive sites were found empirically, we can be more confident about their labels.

To remove duplicate samples, we applied the first and second procedures to both training and validation samples. The specific details of these procedures are outlined in Tables 2 and 3 (Steps 1 and 2). Additionally, figure S1 in the Additional file 1 displays the graph depicting the relationship between the number of samples and window sizes. After creating windows from the test set of proteins, we did not remove duplicate samples. This is because the proteins in the test set are intended to represent real-world applications. In fact, the presence of duplicate fixed-sized samples is a limitation of ML or pre-processing methods and not a characteristic of the actual data, e.g., raw protein sequences. Another important reason to consider is the need for a fair and accurate comparison between various window sizes and methods. Since window sizes directly impact the number of duplicated samples, comparing methods with dissimilar sample numbers would not yield the correct results.

**Table 2:** Details of crafting different window sizes on the training set

Window size	Samples with duplicate sequences and labels (Type 1)			Samples with duplicate sequences (Type 2)			Samples after removing types 1 and 2		
	All	Positive samples	Negative samples	All	Positive samples	Negative samples	All	Positive samples	Negative samples
5	143,420	3728	139,692	154,835	12,125	142,710	84,861	13,122	71,739
7	6556	213	6343	7150	517	6633	191,493	15,066	176,427
9	2619	134	2485	2728	191	2537	193,891	15,106	178,785
15	1582	87	1495	1623	109	1514	194,544	15,133	179,411
21	1336	72	1264	1361	85	1276	194,699	15,141	179,558
27	1212	60	1152	1233	71	1162	194,776	15,147	179,629
33	1152	54	1098	1136	59	1077	194,829	15,151	179,678
45	1025	48	977	1034	53	981	194,888	15,153	179,735
55	969	44	925	978	49	929	194,920	15,155	179,765
77	854	37	817	863	42	821	194,992	15,159	179,833
99	771	34	737	776	39	737	195,047	15,161	179,886

Two types of duplication appeared in the data: Type1: identical sequences and labels; Type 2: identical sequences but different labels

**Table 3:** Details of crafting different window sizes on the validation set. Two types of duplication appeared in the data: Type1: identical sequences and labels; Type 2: identical sequences but different labels

Window size	Samples with duplicate sequences and labels (Type 1)			Samples with duplicate sequences (Type 2)			Samples after removing type 1 and 2		
	All	Positive samples	Negative samples	All	Positive samples	Negative samples	All	Positive samples	Negative samples
5	4861	47	4814	5492	398	5094	17,547	1589	15,958
7	172	6	166	180	10	170	20,506	1610	18,896
9	97	0	97	99	1	98	20,548	1613	18,935
15	57	0	57	59	1	58	20,571	1613	18,958
21	46	0	46	48	1	47	20,578	1613	18,965
27	42	0	42	44	1	43	20,580	1613	18,967
33	32	0	32	34	1	33	20,585	1613	18,972
45	20	0	20	20	0	20	20,592	1613	18,979
55	12	0	12	12	0	12	20,596	1613	18,983
77	8	0	8	8	0	8	20,598	1613	18,985
99	2	0	2	2	0	2	20,601	1613	18,988

PTMs can occur at specific sites within the 3D structure of a protein, and therefore, the structural context of a site can be informative for predicting its likelihood of being modified [60–62]. Given recent breakthroughs in predicting functional and structural protein properties using raw protein sequences [63–65], we can infer that predicting Ubi-sites by shortening the number of amino acids in the windows could result in a lower amount of implicit structural features that ML methods could obtain during training, compared to longer window sizes. By decreasing the length of window sizes, it can be argued that the upper threshold for achieving the best performance would potentially decrease due to the limitation of information in fixed-size window samples. We can argue that by decreasing the length of window sizes, the upper threshold for reaching the best performance would potentially decrease as a consequence of limiting the information in fixed-size window samples.

### Prepare features

The preparation of features for ML models requires individual handling of each feature, which is described in the following.

*PSSM* To ensure a consistent sample size, padding characters are transformed into zero vectors at the end of each window in the PSSM feature. It is crucial to note that in preparing this feature for ML models, all 20 features for each amino acid are taken into account, resulting in the transformation of each amino acid into a 20-dimensional vector.

*PCP* This feature involves the conversion of each amino acid in fixed-sized windows into a fixed-sized vector with a size of 16. To ensure a fixed sample size, padding characters are transformed into zero vectors.

*DPC* It involves the conversion of each fixed-sized window into a fixed-sized vector with 400 values (20 × 20). Padding characters are not needed for this feature.

*AAC* It involves the conversion of each fixed-sized window, ranging from 5 to 99, into a fixed-sized vector with 20 values. Padding characters are not needed for this feature.

## Experiments

In this section, we conducted extensive experiments to predict Ubi-sites. The results are divided into three parts based on the types of features and classification models:

*Feature-based conventional ML methods* Only features were used as input to predict Ubi-sites in this section. Conventional ML methods were used for this purpose.

*End-to-end sequence-based DL methods* Only sequences of amino acids were used as input in this section. DNN architectures were used for this purpose.

*Hybrid feature-based DL methods* Both engineered and sequence-based features were concatenated together as inputs in this section. Similar to the sequence-based type, only DNN architectures were used for this purpose.

It is notable that the baseline result in terms of macro-F1 score is 0.305 if we have a random classifier.

### Feature-based conventional ML methods

All the experiments in this section were conducted using the Scikit-learn [66] framework in Python. It should be noted that all methods were run five times with different random seeds to ensure the robustness of the results.

In this section, we used PSSM, AAC, DPC, and PCP features to predict Ubi-sites using XGBoost, SVM, KNN, and RF methods. All engineered features were reshaped into vectors and fed into the models. To train a model with the best hyperparameters, we used a grid search for kernel, gamma, and C in SVM. In the end, the kernel was RBF, C was in the range of 0.1–100 (with 0.1, 0.5, 1, 10, 100 values), and the gamma range was 0.0001–1 (0.0001, 0.001, 0.01, 0.1). In addition, we used DNN with 3 layers of 128, 64, and 2 nodes that were connected to a softmax layer. For training, we used a learning rate of 0.001 with cosine learning rate decay, weight decay of 1.2e−6, grad clip 5, and 80 epochs.

For KNN, we used neighbors in the range of 3 to 9, while the RF model was trained with max depth values in the range of 6 to 12. To find the best parameters, we used a greedy search algorithm. All results based on the macro-F1 score are shown in Table 4. The result of the best-performing model for each feature appears in bold. The evaluation of different ML approaches was conducted for window sizes of 9, 15, and 21. The KNN model achieved superior results when the AAC feature was utilized at a window size of 9 (0.507). The best F1-score for the XGboost was obtained for the PSSM feature at a window size of 15 (0.468); the RF classifier attained the best result when applied to the AAC feature at a window size of 21 (0.459); and the SVM method aligned more effectively with the DPC feature at a window size of 15 (0.508). Lastly, the DNN method exhibited better performance when combined with the physicochemical feature at a window size 21 (0.537). Overall, DNN is the best model in terms of macro-F1 score. The detailed results for each feature with all tested window sizes are presented in the Additional file 1: Tables S1–S4. Additionally, the AAC feature had the lowest accuracy when used with DNN. Furthermore, it is discernible that, predominantly, a window size encompassing 21 amino acids mostly tends to attain its peak in relation to the macro F1-score metric.

**Table 4:** Comparison of feature-based results based on the macro-F1 score on various window sizes. For each classification model, the best results are tabulated in bold

Window size	Feature	Method				
		KNN	XGBoost	RF	SVM	DNN
9	PSSM	0.441	0.451	0.439	0.450	0.520
	AAC	**0.507**	0.437	0.435	0.416	0.489
	DPC	0.491	0.435	0.435	0.506	0.518
	Physicochemical	0.422	0.460	0.429	0.46	0.516
15	PSSM	0.454	**0.468**	0.440	0.441	0.526
	AAC	0.446	0.462	0.452	0.435	0.503
	DPC	0.458	0.462	0.452	**0.508**	0.523
	Physicochemical	0.423	0.466	0.430	0.467	0.533
21	PSSM	0.440	0.452	0.440	0.419	0.523
	AAC	0.447	0.450	**0.459**	0.441	0.500
	DPC	0.438	0.457	0.450	0.488	0.523
	Physicochemical	0.425	0.462	0.431	0.445	**0.537**

### End-to-end sequence-based DL methods

In this section, we used two Nvidia RTX 2070 GPUs to train the models. All the experiments were conducted in Python using the PyTorch [67] framework. We used the AdaBelief [68] optimizer in conjunction with the mixed precision [69] technique to train models. It is worth noting that we trained all architectures five times, each with different random seeds.

Below are the details of each architecture used in the following experiments.

*LSTM* We used two layers of bidirectional LSTM [43] with 32 units for each layer. Moreover, using the embedding layer, strings of amino acids were converted to a learnable feature with a size of 256. We also constructed a larger bidirectional LSTM model with two layers and 128 units to assess the impact of increased parameters.

*BERT-small* We used the exact architecture reported in the paper [44], except we built the architecture using 8 BERT-base-uncased blocks. Furthermore, the model (embedding) size was 768.

*BERT-tiny* We changed the architecture of the BERT-base reported in the paper by defining 8 BERT-base-uncased blocks, 8 attention heads, and 768 and 320 as the dimensions of the feed-forward layer and model (embedding) size, respectively.

*Nystromformer* We used the exact architecture reported in the paper, except we built the architecture using six Nystromformer blocks. Furthermore, the model (embedding) size was 768.

*SqueezeBERT* We used the exact architecture reported in the paper, which includes 12 transformer blocks. In this architecture, the model (embedding) size was 768.

The details of hyperparameters for each architecture are listed in Table 5.

**Table 5:** Training hyperparameters for both end-to-end and hybrid methods

Architecture	LSTM	BERT-small	BERT-tiny	Nystromformer	SqueezeBERT
Learning rate	8e−04	8e−05	8e−05	5e−05	1e−04
Warmup steps	1000	1000	600	1000	1000
Scheduler	Cosine	Cosine	Cosine	Cosine	Cosine
Decouple weight decay	False	True	True	True	True
Weight decay	1.2e−06	1e−03	1e−04	1e−02	1e−02
Batch size	512	512	512	512	512
Gradient clip	5	2	5	2	2
Label smoothing	0.0	0.2	0.0	0.1	0.1
Mixed precision	True	True	True	True	True

Since we were working with an imbalanced dataset, we adopted two strategies to address this challenge. Firstly, we implemented a weighted loss function, which assigned greater weight to positive labels. In the second approach, we selected all positive samples and randomly chose a subset of negative samples based on the number of positive samples and their locations within the sequences. Specifically, we selected all negative samples that were at least 50 amino acids away from positive sites. By utilizing these strategies, we effectively mitigated the issue of imbalanced labels in our analysis.

In this section, we used only the sequences of amino acids as input for our models. To accomplish this, we employed the word2vec technique [70] to convert the amino acid characters into learnable embedding vectors.

**Fig. 5:**
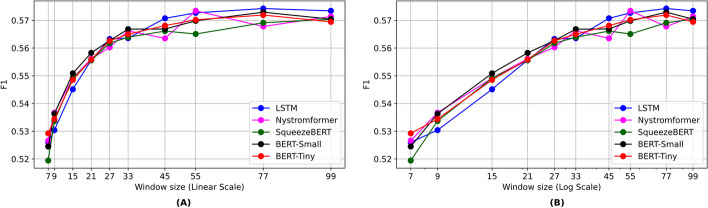
Sequence-based methods using a weighted loss strategy. **A** linear scale. **B** log scale

The results of different architectures on various window sizes, based on the weighted loss function approach to address imbalanced data, are presented in Fig. 5 and Table 6. The difference between BERT-small and BERT-tiny was negligible. SqueezeBERT performed the worst among all the models. Interestingly, despite having fewer parameters, the LSTM model showed the best performance on larger window sizes. The detailed results of different architectures on various window sizes in terms of macro precision, recall, and accuracy are given in the Additional file 1: Tables S5–S7.

**Table 6:** Sequence-based results based on the macro-F1 score on the test set using a weighted loss strategy. The bold values show the best results

Window size	LSTM	BERT-small	BERT-tiny	Nystromformer	SqueezeBERT
5	0.532 ± 0.003	0.543 ± 0.013	0.541 ± 0.001	0.543 ± 0.001	0.545 ± 0.001
7	0.526 ± 0.002	0.524 ± 0.005	0.529 ± 0.002	0.527 ± 0.001	0.519 ± 0.007
9	0.53 ± 0.001	0.536 ± 0.001	0.534 ± 0.001	0.536 ± 0.003	0.533 ± 0.002
15	0.545 ± 0.001	0.55 ± 0.001	0.548 ± 0.002	0.549 ± 0.001	0.549 ± 0.002
21	0.555 ± 0.003	0.558 ± 0.002	0.555 ± 0.002	0.556 ± 0.003	0.556 ± 0.003
27	0.563 ± 0.003	0.562 ± 0.002	0.562 ± 0.003	0.56 ± 0.002	0.561 ± 0.002
33	0.563 ± 0.004	0.566 ± 0.004	0.564 ± 0.002	0.566 ± 0.003	0.563 ± 0.002
45	0.571 ± 0.002	0.566 ± 0.003	0.568 ± 0.002	0.563 ± 0.006	0.566 ± 0.001
55	0.572 ± 0.003	0.569 ± 0.005	0.57 ± 0.003	0.573 ± 0.005	0.565 ± 0.002
77	**0.574 ± 0.004**	0.572 ± 0.003	0.571 ± 0.003	0.567 ± 0.01	0.569 ± 0.004
99	0.573 ± 0.002	0.57 ± 0.004	0.596 ± 0.004	0.571 ± 0.007	0.57 ± 0.001
Avg (5–99)	0.555 ± 0.009	0.556 ± 0.017	0.558 ± 0.008	0.556 ± 0.015	0.554 ± 0.01
Avg (7–99)	0.557 ± 0.008	0.557 ± 0.011	0.56 ± 0.008	0.557 ± 0.015	0.555 ± 0.01

We also observed that macro-F1 values were higher for the validation set compared to the test set, highlighting the importance of having an independent test set, like the one we provided, to assess the generalization performance of the models.

Based on our preliminary experimental results, it was observed that the first strategy handling imbalanced data (weighted loss) performed slightly better in terms of the average macro-F1 metric across all window sizes. The sample results of the second approach (balanced sample strategy) for the BERT-small model are presented in Table 7.

**Table 7:** Sequence-based results based on the macro-F1 score on the test set using the balanced sample strategy. The bold values show the best results

Window size	BERT-small
5	0.548 ± 0.009
7	0.523 ± 0.01
9	0.533 ± 0.001
15	0.541 ± 0.0
21	0.552 ± 0.002
27	0.565 ± 0.002
33	0.571 ± 0.003
45	0.570 ± 0.006
55	**0.571 ± 0.002**
77	0.571 ± 0.004
99	0.569 ± 0.005
Avg (5–99)	0.555 ± 0.005
Avg (7–99)	0.556 ± 0.006

Our results demonstrate that DL methods outperformed classical ML methods in terms of accuracy and F1-score. The best macro-F1 score of 0.574 was achieved using LSTM with a window size of 77, as shown in bold in Table 6.

### Hybrid feature-based DL methods

In this section, we concatenated both the features and embedding of amino acid sequences and fed them to the neural networks. We employed the weighted-loss strategy for this section.

As in Sect. "End-to-end sequence-based DL methods", the LSTM architecture demonstrated superior performance with regard to sequences; consequently, we used only the LSTM model for training purposes to facilitate a more straightforward comparison in terms of computational cost. An additional advantage of using LSTM is that there are fewer parameters, which makes it much more efficient in terms of computational cost.

To create hybrid features, we combined the PSSM and PCP features with the output of the embedding layer, taking into account the number of amino acids. To incorporate the AAC and DPC features, we replicated the fixed features in proportion to the number of amino acids within the specified window size. We then merged these replicated features with the output of the embedding layer in a similar manner to the PSSM and PCP features.

**Fig. 6:**
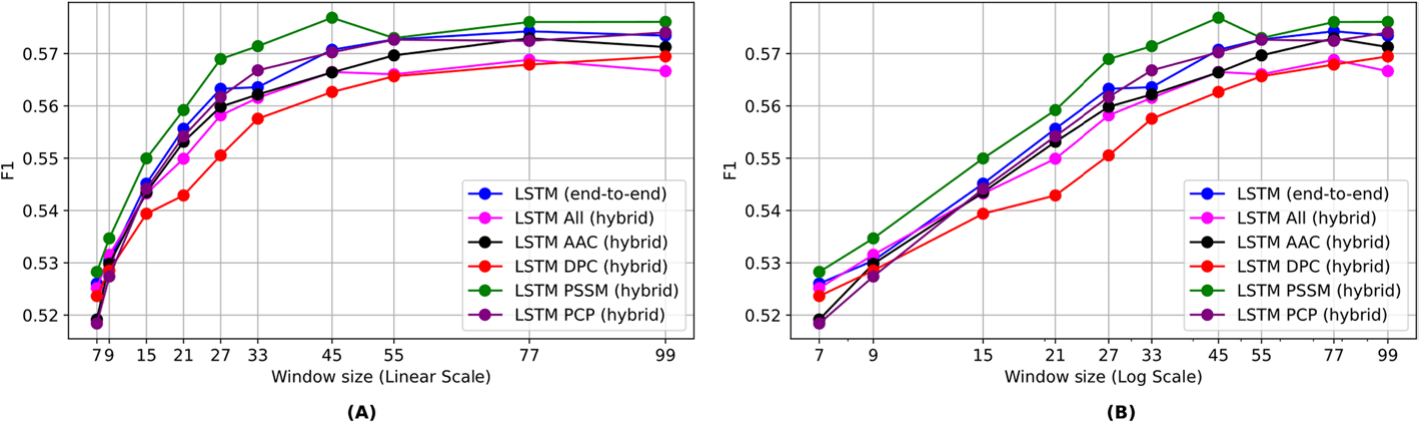
Hybrid feature-based methods. **A** linear scale. **B** log scale

The results in terms of the macro-F1 score are placed in Table 8 and plotted in Fig. 6. The detailed results in terms of macro precision, recall, and accuracy are shown in the Additional file 1: Tables S8–S10, while the best results achieved in terms of all introduced evaluation metrics are summarized in Table 9. Furthermore, the results of the larger LSTM model are placed in the Additional file 1: Figure S2.

**Table 8:** Hybrid results for the LSTM model based on the macro-F1 score on the test set. The bold values show the best results

Window size	Seq	Seq + PSSM	Seq + AAC	Seq + DPC	Seq + PCP	Seq + All
5	0.530 ± 0.003	0.518 ± 0.003	0.490 ± 0.001	0.502 ± 0.001	0.499 ± 0.019	0.522 ± 0.002
7	0.520 ± 0.002	0.528 ± 0.004	0.519 ± 0.002	0.523 ± 0.001	0.518 ± 0.01	0.525 ± 0.002
9	0.530 ± 0.001	0.534 ± 0.002	0.529 ± 0.001	0.528 ± 0.002	0.527 ± 0.006	0.531 ± 0.001
15	0.545 ± 0	0.55 ± 0.005	0.543 ± 0.002	0.539 ± 0.002	0.544 ± 0.006	0.543 ± 0.003
21	0.555 ± 0.003	0.559 ± 0.003	0.553 ± 0.002	0.542 ± 0.001	0.554 ± 0.003	0.549 ± 0.005
27	0.563 ± 0.003	0.568 ± 0.001	0.559 ± 0.002	0.551 ± 0.002	0.562 ± 0.002	0.558 ± 0.007
33	0.563 ± 0.004	0.571 ± 0.002	0.562 ± 0.002	0.557 ± 0.001	0.569 ± 0.004	0.561 ± 0.006
45	0.570 ± 0.002	0.576 ± 0.007	0.566 ± 0.002	0.562 ± 0.002	0.570 ± 0.003	0.566 ± 0.003
55	0.572 ± 0.003	0.572 ± 0.006	0.569 ± 0.002	0.565 ± 0.004	0.573 ± 0.003	0.566 ± 0.002
77	0.574 ± 0.004	0.576 ± 0.004	0.572 ± 0.003	0.567 ± 0.004	0.572 ± 0.003	0.569 ± 0.003
99	0.573 ± 0.002	**0.576 ± 0.002**	0.571 ± 0.002	0.569 ± 0.003	0.574 ± 0.002	0.566 ± 0.002
Avg (5–99)	0.554 ± 0.009	0.557 ± 0.013	0.548 ± 0.006	0.546 ± 0.008	0.551 ± 0.025	0.551 ± 0.013
Avg (7–99)	0.557 ± 0.008	0.561 ± 0.013	0.554 ± 0.006	0.550 ± 0.008	0.556 ± 0.016	0.553 ± 0.013

**Table 9:** Best hybrid results for the LSTM model on the test set. The bold values show the best results

Features				Metrics						Window size
PSSM	AAC	DPC	PCP	Accuracy	Macro precision	Macro recall	Macro-F1	F1^positive^	MCC	
–	–	–	–	82.717 ± 0.917	0.57 ± 0.001	0.581 ± 0.011	0.574 ± 0.004	0.9 ± 0.003	0.396	77
■	–	–	–	**81.984 ± 2.028**	**0.572 ± 0.005**	**0.59 ± 0.023**	**0.576 ± 0.007**	**0.902 ± 0.005**	**0.402**	**45**
–	■	–	–	82.036 ± 1.282	0.568 ± 0.001	0.583 ± 0.011	0.572 ± 0.003	0.896 ± 0.004	0.398	77
–	–	■	–	82.73 ± 0.008	0.566 ± 0.002	0.574 ± 0.005	0.569 ± 0.003	0.897 ± 0.003	0.396	99
–	–	–	■	82.006 ± 1.026	0.568 ± 0.003	0.584 ± 0.005	0.574 ± 0.002	0.901 ± 0.002	0.397	99
■	■	■	■	83.13 ± 0.658	0.567 ± 0.002	0.572 ± 0.007	0.569 ± 0.003	0.889 ± 0.002	0.395	77

(■) When combined with the sequence feature

(–) When not combined with sequence feature

Based on the results presented in Table 9, incorporating a combination of raw amino acid sequences and handcrafted features generally resulted in a slight improvement in LSTM model performance. The highest macro-F1 score of 0.576 was achieved with a window size of 45 and utilizing PSSM as the feature in combination with raw sequences. The F1 score, precision, and recall values of the positive class for a window size of 45 were 0.902, 0.8786, and 0.9147, respectively. Furthermore, as expected, the window size 45 exhibits the best MCC value of 0.402.

## Discussion

Ubiquitination is a critical PTMs that regulates many important cellular processes in humans. Taking into consideration the significance of the topic, there arises a fundamental requirement for cost- and time-effective alternatives to traditional Ubi-site detection. Presently, no practical computational tool is available to accurately predict Ubi-sites in human data. In this work, we conducted extensive experiments to evaluate the performance of both classical ML techniques and end-to-end DL methods for predicting Ubi-sites in human proteins. To facilitate fair comparisons between the predicted models, we designed a benchmark with open-access datasets (collected from the dbPTM 2019 and dbPTM 2022 databases), standard evaluation metrics, and the proper validation strategy to avoid any potential information leakage.

We conducted a rigorous empirical comparison of ten different approaches across three paradigms: feature-based conventional ML approaches, end-to-end sequence-based DL models, and hybrid feature-based DL methods. The primary aim was to establish robust baselines that can guide future model development and facilitate meaningful comparisons.

Our results demonstrated that DL approaches outperformed traditional ML techniques across various evaluation metrics, specifically achieving a macro-F1 score of 0.574 compared to 0.537. Specifically, in contrast to many customized-designed DL network architectures [71], we found that the LSTM architecture achieved the highest macro-F1 score using only raw amino acid sequences as input. This finding is consistent with previous research [14, 28] and shows the capability of DNN architectures to automatically learn meaningful features from protein sequences for ubiquitination site prediction.

Furthermore, incorporating hand-crafted features along with raw sequences typically led to a marginal enhancement compared to using raw sequences alone. Specifically, there was a slight increase in the macro-F1 score, with values of 0.576 and 0.574, respectively. This suggests that hand-crafted features may provide useful biological insights that complement the representations learned by deep networks. Similar to a Ubi-site study [30], we also observed that longer window sizes led to better performance, likely due to the additional context they provide about the local protein environment. Overall, our findings highlight the potential of DL techniques for ubi-site prediction and the importance of both input features and window size in model design.

## Conclusion

This work demonstrates the capability of ML techniques, particularly DL approaches, in accurately predicting Ubi-sites in human proteins. Through extensive experiments, we observed that end-to-end DNNs outperformed conventional ML methods across various evaluation metrics. Notably, LSTM architectures achieved the best results when utilizing raw amino acid sequences, highlighting their ability to automatically extract meaningful features. The success of LSTM architectures in such bioinformatics classification problems can be attributed to their ability to capture long-term dependencies in sequential data, their robustness to noisy data, and their capacity for representation learning. Additionally, incorporating engineered features like PPSM in combination with the raw sequences resulted in a slight improvement in model performance.

Our analysis further revealed that larger window sizes contain more contextual information that facilitates better prediction. Overall, these findings showcase the tremendous potential of ML methods for unraveling the mechanisms of post-translational protein regulation through ubiquitination.

The creation of a standardized human ubiquitination benchmark dataset is a significant contribution to this work. By splitting proteins from two releases of dbPTM into training, validation, and independent test sets, we enabled direct comparison between old and new data. Importantly, our study contributes to enhancing reproducibility and enabling fair comparisons among various proposals for the Ubi-site prediction task. To ensure transparency and reproducibility, we shared the datasets and code used in our study, allowing other researchers to replicate and build upon our findings. The availability of these resources promotes a standardized evaluation framework for various Ubi-site estimation approaches. In summary, by harnessing advanced ML algorithms and benchmark datasets, we can gain a deeper understanding of ubiquitination patterns and their influence on cellular processes. The methodologies developed here will assist in elucidating the intricacies of protein regulation and hold promising implications for biomedical applications.

### Supplementary Information

**Additional file 1. Figure S1: ** Number of both positive and negative samples in Set 1. (A) positive, (B) negative, and (C) both positive and negative. **Table S1:** PSSM feature results based on the macro-F1 score on the test set. **Table S2:** PCP feature results based on the macro-F1 score on the test set. **Table S3:** AAC feature results based on the macro-F1 score on the test set. **Table S4:** DPC features results based on the macro-F1 score on the test set. **Table S5:** Sequence-based results based on macro recall metric on the test set using weighted loss strategy. **Table S6:** Sequence-based results based on macro precision metric on the test set using weighted loss strategy. **Table S7:** Sequence-based results based on accuracy metric on the test set using weighted loss strategy. **Table S8:** Hybrid results based on macro precision metric on the test set. **Table S9:** Hybrid results based on macro recall metric on the test set. **Table S10:** Hybrid results based on accuracy metric on the test set. **Figure S2:** The difference between large and small LSTM models in Hybrid-based and sequence-based methods Macro-F1 score on the test set. (A) linear scale. (B) log scale.

## Data Availability

The data and source code are available in the public repository: https://github.com/mahdip72/ubi.

## Abbreviations

PTMs: Post-translational modifications
ML: Machine learning
MS: Mass spectrometry
DUBs: De-ubiquitylating enzymes
PLA: Proximity ligation assay
IP: Immunoprecipitation
DL: Deep learning
RF: Random forest
XGB: Extreme gradient boosting
SVM: Support vector machine
KNN: K-nearest neighbor
LR: Logistic regression
EBMC: Efficient Bayesian Multivariate Classifier
AAC: Amino acid composition
CNN: Convolutional neural network
BE: Binary encoding
PseAAC: Pseudo-amino acid composition
CKSAAP: Composition of k-spaced amino acid pairs
PSPM: Position-specific propensity matrices
DNNs: Deep neural networks
ANNs: Artificial neural networks
RNN: Recurrent neural networks
LSTM: Long short-term memory
BERT: Bidirectional encoder representation transformer
NAS: Neural architecture search
DPC: Dipeptide composition
PCP: Physicochemical properties
PSSM: Position-specific scoring matrices
HUSB: Human ubiquitination sites benchmark

## Footnotes

1 O(n) describes an algorithm whose runtime grows linearly with the size of the input dataset, meaning that as the number of items in the input increases, the time it takes to complete the algorithm will also increase at a steady rate.

2 A hold-out dataset is a subset of a larger dataset that is separated from the main dataset and used to evaluate the performance of a machine learning model. The hold-out dataset is not used for training the model but is instead held aside and used to test the model's ability to make predictions on new data. Using a hold-out dataset allows for a more accurate assessment of the model's generalization performance, as it ensures that the model has not been overfitted to the training data.
