# Supplementary material for: Machine learning-based approaches for ubiquitination site prediction in human proteins
Source: BMC Bioinformatics. 2023 Nov 28;24:449. doi: 10.1186/s12859-023-05581-w (PMC10683244; doi:10.1186/s12859-023-05581-w)
Supplement: Supplementary file 1 — Additional file 1. Figure S1: Number of both positive and negative samples in Set 1. (A) positive, (B) negative, and (C) both positive and negative. Table S1: PSSM feature results based on the macro-F1 score on the test set. Table S2: PCP feature results based on the macro-F1 score on the test set. Table S3: AAC feature results based on the macro-F1 score on the test set. Table S4: DPC features results based on the macro-F1 score on the test set. Table S5: Sequence-based results based on macro recall metric on the test set using weighted loss strategy. Table S6: Sequence-based results based on macro precision metric on the test set using weighted loss strategy. Table S7: Sequence-based results based on accuracy metric on the test set using weighted loss strategy. Table S8: Hybrid results based on macro precision metric on the test set. Table S9: Hybrid results based on macro recall metric on the test set. Table S10: Hybrid results based on accuracy metric on the test set. Figure S2: The difference between large and small LSTM models in Hybrid-based and sequence-based methods Macro-F1 score on the test set. (A) linear scale. (B) log scale. [file 12859_2023_5581_MOESM1_ESM.docx]

Machine Learning-Based Approaches for Ubiquitination Site Prediction in Human Proteins

Mahdi Pourmirzaei ^1^, Shahin Ramazi^2^, Farzaneh Esmaili^1^, Seyedehsamaneh Shojaeilangari^3,*^, Abdollah Allahvardi^2^

*^1 Department of Information Technology, Tarbiat Modares University^*^,^ *^14115-111, Tehran, Iran^*

*^2 Department of Biophysics, Faculty of Biological Sciences, Tarbiat Modares University, 14115-111, Tehran, Iran^*

*^3 Biomedical Engineering group, Department of Electrical and Information Technology, Iranian Research Organization (IROST), 33535111, Tehran, Iran^*

^*^ *Corresponding author: Seyedehsamaneh Shojaeilangari, E-mail: s.shojaie@irost.ir*

# Dataset Supplementary Information

The number of both positive and negative samples based on different window lengths in Set 1 of the human ubiquitination site benchmark (HUSB) after removing duplicated samples is depicted in figure S1.


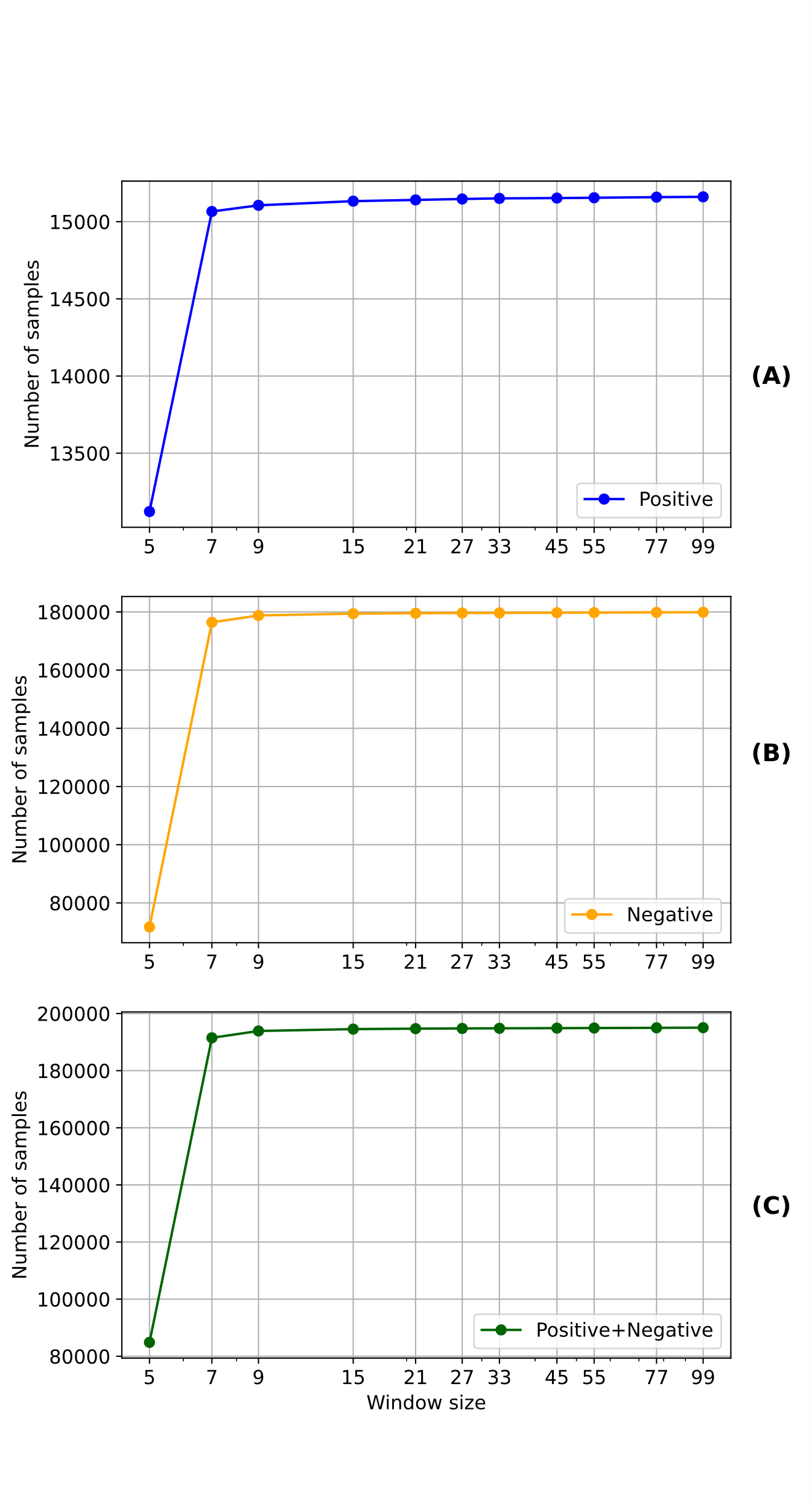


Figure S1: Number of both positive and negative samples in Set 1. (A) positive, (B) negative, and (C) both positive and negative.

# Supplementary results

In this section, we included our additional results divided into three parts based on the types of features and classification models:

## Feature-based ML methods

Tables S1-S4 show the classification results of PSSM, PCP, AAC, and DPC features respectively with different window sizes in terms of macro-F1 score.

Table S1: PSSM feature results based on the macro-F1 score on the test set.

| **Window size** | **KNN** | **XGBoost** | **Random Forest** | **SVM** | **DNN** |
| --- | --- | --- | --- | --- | --- |
| 5 | 0.441 | 0.442 | 0.436 | 0.448 | 0.514 |
| 7 | 0.443 | 0.446 | 0.438 | 0.449 | 0.517 |
| 9 | 0.441 | 0.451 | 0.439 | 0.450 | 0.520 |
| 15 | 0.454 | 0.468 | 0.440 | 0.441 | 0.526 |
| 21 | 0.440 | 0.452 | 0.440 | 0.419 | 0.523 |
| 27 | 0.436 | 0.449 | 0.439 | 0.392 | 0.526 |
| 33 | 0.438 | 0.437 | 0.436 | 0.363 | **0.527** |

Table S2: PCP feature results based on the macro-F1 score on the test set.

| **Window size** | **KNN** | **XGBoost** | **Random Forest** | **SVM** | **DNN** |
| --- | --- | --- | --- | --- | --- |
| 5 | 0.436 | 0.453 | 0.412 | 0.446 | 0.478 |
| 7 | 0.431 | 0.458 | 0.424 | 0.450 | 0.501 |
| 9 | 0.422 | 0.460 | 0.429 | 0.460 | 0.516 |
| 15 | 0.423 | 0.466 | 0.430 | 0.467 | 0.533 |
| 21 | 0.425 | 0.462 | 0.431 | 0.445 | 0.537 |
| 27 | 0.428 | 0.460 | 0.434 | 0.441 | **0.538** |
| 33 | 0.432 | 0.458 | 0.433 | 0.439 | 0.536 |

Table S3: AAC feature results based on the macro-F1 score on the test set.

| **Window size** | **KNN** | **XGBoost** | **Random Forest** | **SVM** | **DNN** |
| --- | --- | --- | --- | --- | --- |
| 5 | **0.508** | 0.416 | 0.417 | 0.41 | 0.46 |
| 7 | 0.487 | 0.435 | 0.434 | 0.425 | 0.482 |
| 9 | 0.507 | 0.437 | 0.435 | 0.416 | 0.489 |
| 15 | 0.446 | 0.462 | 0.452 | 0.435 | 0.503 |
| 21 | 0.447 | 0.450 | 0.459 | 0.441 | 0.500 |
| 27 | 0.431 | 0.449 | 0.464 | 0.450 | 0.496 |
| 33 | 0.440 | 0.450 | 0.462 | 0.452 | 0.499 |

Table S4: DPC features results based on the macro-F1 score on the test set.

| **Window size** | **KNN** | **XGBoost** | **Random Forest** | **SVM** | **DNN** |
| --- | --- | --- | --- | --- | --- |
| 5 | 0.508 | 0.417 | 0.412 | 0.499 | 0.458 |
| 7 | 0.509 | 0.430 | 0.425 | 0.500 | 0.506 |
| 9 | 0.491 | 0.435 | 0.435 | 0.506 | 0.518 |
| 15 | 0.458 | 0.462 | 0.452 | 0.508 | **0.523** |
| 21 | 0.438 | 0.457 | 0.450 | 0.488 | **0.523** |
| 27 | 0.428 | 0.449 | 0.467 | 0.467 | 0.521 |
| 33 | 0.431 | 0.451 | 0.464 | 0.452 | 0.519 |

## End-to-end sequence-based methods

The results of different architectures on various window sizes in terms of macro recall, precision, and accuracy are presented in tables S1-S7 respectively.

Table S5: Sequence-based results based on macro recall metric on the test set using weighted loss strategy.

| **Window Size** | **LSTM** | **BERT-small** | **BERT-tiny** | **Nystromformer** | **SqueezeBERT** |
| --- | --- | --- | --- | --- | --- |
| 5 | 0.565 ± 0.002 | 0.562 ± 0.002 | 0.562 ± 0.002 | 0.545 ± 0.002 | 0.56 ± 0.002 |
| 7 | 0.533 ± 0.001 | 0.534 ± 0.003 | 0.534 ± 0.003 | 0.528 ± 0.002 | 0.528 ± 0.014 |
| 9 | 0.533 ± 0.003 | 0.537 ± 0.003 | 0.537 ± 0.003 | 0.543 ± 0.001 | 0.542 ± 0.003 |
| 15 | 0.551 ± 0.004 | 0.551 ± 0.003 | 0.551 ± 0.003 | 0.556 ± 0.006 | 0.554 ± 0.006 |
| 21 | 0.566 ± 0.004 | 0.558 ± 0.002 | 0.558 ± 0.002 | 0.564 ± 0.007 | 0.56 ± 0.007 |
| 27 | 0.575 ± 0.01 | 0.567 ± 0.008 | 0.567 ± 0.008 | 0.559 ± 0.005 | 0.563 ± 0.004 |
| 33 | 0.57 ± 0.007 | 0.566 ± 0.004 | 0.566 ± 0.004 | 0.57 ± 0.008 | 0.567 ± 0.008 |
| 45 | 0.579 ± 0.007 | 0.57 ± 0.006 | 0.57 ± 0.006 | 0.561 ± 0.01 | 0.567 ± 0.004 |
| 55 | **0.583 ± 0.012** | 0.575 ± 0.008 | 0.575 ± 0.008 | 0.576 ± 0.007 | 0.563 ± 0.002 |
| 77 | 0.581 ± 0.011 | 0.575 ± 0.008 | 0.575 ± 0.008 | 0.568 ± 0.017 | 0.57 ± 0.009 |
| 99 | 0.582 ± 0.013 | 0.569 ± 0.008 | 0.569 ± 0.008 | 0.573 ± 0.012 | 0.573 ± 0.004 |
| Avg (5 to 99) | 0.565 ± 0.026 | 0.56 ± 0.019 | 0.56 ± 0.019 | 0.558 ± 0.028 | 0.559 ± 0.022 |
| Avg (7 to 99) | 0.565 ± 0.026 | 0.56 ± 0.018 | 0.56 ± 0.018 | 0.56 ± 0.028 | 0.559 ± 0.022 |

Table S6 Sequence-based results based on macro precision metric on the test set using weighted loss strategy.

| **Window Size** | **LSTM** | **BERT-small** | **BERT-tiny** | **Nystromformer** | **SqueezeBERT** |
| --- | --- | --- | --- | --- | --- |
| 5 | 0.541 ± 0.002 | 0.552 ± 0.012 | 0.543 ± 0.001 | 0.543 ± 0.001 | 0.544 ± 0.001 |
| 7 | 0.525 ± 0.001 | 0.525 ± 0.003 | 0.528 ± 0.002 | 0.526 ± 0.002 | 0.52 ± 0.007 |
| 9 | 0.529 ± 0.001 | 0.535 ± 0.001 | 0.533 ± 0.002 | 0.534 ± 0.003 | 0.532 ± 0.002 |
| 15 | 0.542 ± 0.001 | 0.551 ± 0.002 | 0.547 ± 0.001 | 0.546 ± 0.001 | 0.546 ± 0.001 |
| 21 | 0.551 ± 0.003 | 0.556 ± 0.002 | 0.554 ± 0.002 | 0.552 ± 0.001 | 0.553 ± 0.001 |
| 27 | 0.558 ± 0.001 | 0.562 ± 0.002 | 0.56 ± 0.002 | 0.563 ± 0.002 | 0.561 ± 0.001 |
| 33 | 0.56 ± 0.002 | 0.566 ± 0.001 | 0.564 ± 0.002 | 0.564 ± 0.001 | 0.562 ± 0.001 |
| 45 | 0.566 ± 0.001 | 0.569 ± 0.001 | 0.567 ± 0.002 | 0.567 ± 0.001 | 0.565 ± 0.003 |
| 55 | 0.568 ± 0.001 | 0.569 ± 0.003 | 0.567 ± 0.002 | 0.57 ± 0.003 | 0.567 ± 0.003 |
| 77 | 0.57 ± 0.001 | 0.571 ± 0.003 | 0.57 ± 0.003 | 0.571 ± 0.002 | 0.569 ± 0.003 |
| 99 | 0.57 ± 0.004 | **0.574 ± 0.003** | 0.571 ± 0.003 | 0.571 ± 0.002 | 0.568 ± 0.002 |
| Avg (5 to 99) | 0.553 ± 0.006 | 0.557 ± 0.014 | 0.555 ± 0.007 | 0.555 ± 0.006 | 0.553 ± 0.009 |
| Avg (7 to 99) | 0.554 ± 0.006 | 0.556 ± 0.007 | 0.556 ± 0.007 | 0.556 ± 0.006 | 0.554 ± 0.009 |

Table S7 Sequence-based results based on accuracy metric on the test set using weighted loss strategy.

| **Window Size** | **LSTM** | **BERT-small** | **BERT-tiny** | **Nystromformer** | **SqueezeBERT** |
| --- | --- | --- | --- | --- | --- |
| 5 | 66.203 ± 0.583 | 74.954 ± 4.055 | 69.26 ± 0.309 | 74.943 ± 0.182 | 70.923 ± 0.216 |
| 7 | 79.017 ± 0.875 | 78.686 ± 3.525 | 79.896 ± 0.757 | 81.22 ± 1.198 | 78.337 ± 2.429 |
| 9 | 81.043 ± 1.123 | 81.432 ± 0.928 | 81.284 ± 1.427 | 80.123 ± 1.46 | 79.373 ± 1.479 |
| 15 | 80.92 ± 1.021 | 82.906 ± 0.846 | 82.288 ± 0.382 | 81.04 ± 1.193 | 81.54 ± 0.978 |
| 21 | 80.653 ± 0.775 | 82.516 ± 1.142 | 82.686 ± 0.403 | 81.367 ± 0.843 | 82.21 ± 0.915 |
| 27 | 81.13 ± 1.46 | 83.14 ± 1.267 | 82.65 ± 1.008 | 83.933 ± 0.715 | 83.15 ± 0.37 |
| 33 | 82.077 ± 0.741 | 83.164 ± 1.266 | 83.286 ± 0.562 | 82.777 ± 1.027 | 82.82 ± 1.163 |
| 45 | 82.133 ± 0.715 | 84.134 ± 0.537 | 83.404 ± 0.89 | 84.31 ± 0.848 | 83.407 ± 0.715 |
| 55 | 82.043 ± 1.299 | 83.506 ± 0.425 | 82.738 ± 0.756 | 83.4 ± 0.987 | 84.107 ± 0.179 |
| 77 | 82.717 ± 0.917 | 83.192 ± 0.896 | 83.256 ± 1.107 | 84.06 ± 1.501 | 83.65 ± 1.186 |
| 99 | 82.44 ± 2.162 | **84.5 ± 0.839** | 83.934 ± 0.954 | 83.727 ± 0.993 | 83.197 ± 0.701 |
| Avg (5 to 99) | 80.034 ± 3.795 | 82.012 ± 6.077 | 81.335 ± 2.801 | 81.9 ± 3.497 | 81.156 ± 3.723 |
| Avg (7 to 99) | 81.417 ± 3.75 | 82.542 ± 4.527 | 82.542 ± 2.784 | 82.596 ± 3.493 | 82.179 ± 3.716 |

## Hybrid feature-based DL methods

The results of the LSTM model on hybrid features in terms of macro recall, precision, and accuracy are presented in tables S8-S10 respectively.

Table S8: Hybrid results based on macro precision metric on the test set.

| **Window Size** | **Seq** | **Seq + PSSM** | **Seq + AAC** | **Seq + DPC** | **Seq + PCP** | **Seq + All** |
| --- | --- | --- | --- | --- | --- | --- |
| 5 | 0.541 ± 0.002 | 0.521 ± 0.002 | 0.517 ± 0 | 0.516 ± 0.001 | 0.518 ± 0.001 | 0.522 ± 0.001 |
| 7 | 0.525 ± 0.001 | 0.528 ± 0.004 | 0.523 ± 0.001 | 0.523 ± 0.002 | 0.526 ± 0.002 | 0.526 ± 0.002 |
| 9 | 0.529 ± 0.001 | 0.532 ± 0.003 | 0.529 ± 0.001 | 0.529 ± 0.002 | 0.53 ± 0.004 | 0.531 ± 0.001 |
| 15 | 0.542 ± 0.001 | 0.547 ± 0.005 | 0.54 ± 0.002 | 0.538 ± 0.001 | 0.544 ± 0.006 | 0.542 ± 0.001 |
| 21 | 0.551 ± 0.003 | 0.556 ± 0.003 | 0.549 ± 0.002 | 0.543 ± 0.003 | 0.55 ± 0.004 | 0.548 ± 0.004 |
| 27 | 0.558 ± 0.001 | 0.565 ± 0.004 | 0.556 ± 0.001 | 0.548 ± 0.002 | 0.558 ± 0.003 | 0.555 ± 0.005 |
| 33 | 0.56 ± 0.002 | 0.567 ± 0.005 | 0.559 ± 0.002 | 0.554 ± 0.001 | 0.562 ± 0.005 | 0.559 ± 0.005 |
| 45 | 0.566 ± 0.001 | 0.572 ± 0.005 | 0.563 ± 0.002 | 0.56 ± 0.001 | 0.567 ± 0.004 | 0.566 ± 0.004 |
| 55 | 0.568 ± 0.001 | 0.571 ± 0.004 | 0.565 ± 0.001 | 0.562 ± 0.002 | 0.57 ± 0.004 | 0.565 ± 0.003 |
| 77 | 0.57 ± 0.001 | 0.573 ± 0.003 | 0.568 ± 0.001 | 0.563 ± 0.003 | 0.567 ± 0.004 | 0.567 ± 0.002 |
| 99 | 0.57 ± 0.004 | 0.572 ± 0.002 | 0.567 ± 0.002 | 0.566 ± 0.002 | 0.568 ± 0.003 | 0.568 ± 0.003 |
| Avg (5 to 99) | 0.553 ± 0.006 | 0.555 ± 0.013 | 0.549 ± 0.005 | 0.546 ± 0.006 | 0.551 ± 0.013 | 0.55 ± 0.011 |
| Avg (7 to 99) | 0.554 ± 0.006 | 0.558 ± 0.012 | 0.552 ± 0.005 | 0.549 ± 0.006 | 0.554 ± 0.013 | 0.553 ± 0.01 |

Table S9: Hybrid results based on macro recall metric on the test set.

| **Window Size** | **Seq** | **Seq + PSSM** | **Seq + AAC** | **Seq + DPC** | **Seq + PCP** | **Seq + All** |
| --- | --- | --- | --- | --- | --- | --- |
| 5 | 0.565 ± 0.002 | 0.533 ± 0.006 | 0.539 ± 0 | 0.532 ± 0.002 | 0.536 ± 0.01 | 0.523 ± 0.003 |
| 7 | 0.533 ± 0.001 | 0.538 ± 0.002 | 0.541 ± 0.001 | 0.525 ± 0.001 | 0.535 ± 0.013 | 0.525 ± 0.002 |
| 9 | 0.533 ± 0.003 | 0.541 ± 0.002 | 0.545 ± 0.001 | 0.529 ± 0.003 | 0.539 ± 0.01 | 0.534 ± 0.004 |
| 15 | 0.551 ± 0.004 | 0.555 ± 0.006 | 0.554 ± 0.004 | 0.544 ± 0.007 | 0.554 ± 0.009 | 0.546 ± 0.007 |
| 21 | 0.566 ± 0.004 | 0.572 ± 0.017 | 0.56 ± 0.002 | 0.545 ± 0.006 | 0.566 ± 0.006 | 0.552 ± 0.007 |
| 27 | 0.575 ± 0.01 | 0.579 ± 0.009 | 0.57 ± 0.009 | 0.555 ± 0.006 | 0.57 ± 0.007 | 0.565 ± 0.012 |
| 33 | 0.57 ± 0.007 | 0.581 ± 0.006 | 0.568 ± 0.001 | 0.565 ± 0.005 | 0.578 ± 0.008 | 0.567 ± 0.011 |
| 45 | 0.579 ± 0.007 | 0.59 ± 0.023 | 0.571 ± 0.004 | 0.566 ± 0.005 | 0.582 ± 0.016 | 0.567 ± 0.003 |
| 55 | 0.583 ± 0.012 | 0.577 ± 0.011 | 0.579 ± 0.007 | 0.574 ± 0.011 | 0.581 ± 0.013 | 0.567 ± 0.005 |
| 77 | 0.581 ± 0.011 | 0.581 ± 0.007 | 0.583 ± 0.011 | 0.578 ± 0.008 | 0.582 ± 0.004 | 0.572 ± 0.007 |
| 99 | 0.582 ± 0.013 | 0.582 ± 0.007 | 0.577 ± 0.005 | 0.574 ± 0.005 | **0.584 ± 0.005** | 0.567 ± 0.007 |
| Avg (5 to 99) | 0.565 ± 0.026 | 0.566 ± 0.035 | 0.562 ± 0.018 | 0.553 ± 0.02 | 0.564 ± 0.033 | 0.553 ± 0.023 |
| Avg (7 to 99) | 0.565 ± 0.026 | 0.57 ± 0.035 | 0.565 ± 0.018 | 0.556 ± 0.02 | 0.567 ± 0.031 | 0.556 ± 0.023 |

Table S10: Hybrid results based on accuracy metric on the test set.

| **Window Size** | **Seq** | **Seq + PSSM** | **Seq + AAC** | **Seq + DPC** | **Seq + PCP** | **Seq + All** |
| --- | --- | --- | --- | --- | --- | --- |
| 5 | 66.203 ± 0.583 | 76.516 ± 3.349 | 66.448 ± 0.202 | 70.962 ± 0.002 | 70.21 ± 6.99 | 81.69 ± 1.098 |
| 7 | 79.017 ± 0.875 | 78.654 ± 2.151 | 74.546 ± 0.379 | 81.098 ± 0.003 | 77.584 ± 7.166 | 82.482 ± 0.712 |
| 9 | 81.043 ± 1.123 | 80.05 ± 0.907 | 77.342 ± 0.185 | 82.092 ± 0.003 | 78.704 ± 4.886 | 81.414 ± 0.98 |
| 15 | 80.92 ± 1.021 | 81.598 ± 0.858 | 79.798 ± 1.051 | 81.25 ± 0.003 | 80.34 ± 3.309 | 81.954 ± 1.416 |
| 21 | 80.653 ± 0.775 | 80.966 ± 2.796 | 81.304 ± 0.348 | 82.18 ± 0.002 | 80.478 ± 1.871 | 82.334 ± 0.452 |
| 27 | 81.13 ± 1.46 | 81.872 ± 1.666 | 81.27 ± 1.344 | 81.944 ± 0.008 | 81.582 ± 1.633 | 81.908 ± 0.75 |
| 33 | 82.077 ± 0.741 | 81.942 ± 1.337 | 82.218 ± 0.421 | 81.43 ± 0.004 | 81.396 ± 1.776 | 82.238 ± 1.288 |
| 45 | 82.133 ± 0.715 | 81.984 ± 2.028 | 82.526 ± 0.387 | 82.75 ± 0.006 | 81.864 ± 2.455 | 83.48 ± 0.572 |
| 55 | 82.043 ± 1.299 | 83.168 ± 0.96 | 81.966 ± 0.985 | 81.91 ± 0.008 | 82.406 ± 2.041 | 83.346 ± 0.709 |
| 77 | 82.717 ± 0.917 | 83.12 ± 0.751 | 82.036 ± 1.282 | 81.744 ± 0.008 | 81.948 ± 0.92 | 83.13 ± 0.658 |
| 99 | 82.44 ± 2.162 | 82.836 ± 0.958 | 82.628 ± 0.697 | 82.73 ± 0.008 | 82.006 ± 1.026 | **83.778 ± 1.102** |
| Avg (5 to 99) | 80.034 ± 3.795 | 81.155 ± 6.025 | 79.28 ± 2.584 | 80.917 ± 0.019 | 79.865 ± 12.508 | 82.523 ± 3.095 |
| Avg (7 to 99) | 81.417 ± 3.75 | 81.619 ± 5.008 | 80.563 ± 2.576 | 81.913 ± 0.018 | 80.831 ± 10.372 | 82.606 ± 2.894 |

The difference between large and small LSTM models in the hybrid mode is plotted in figure S2.


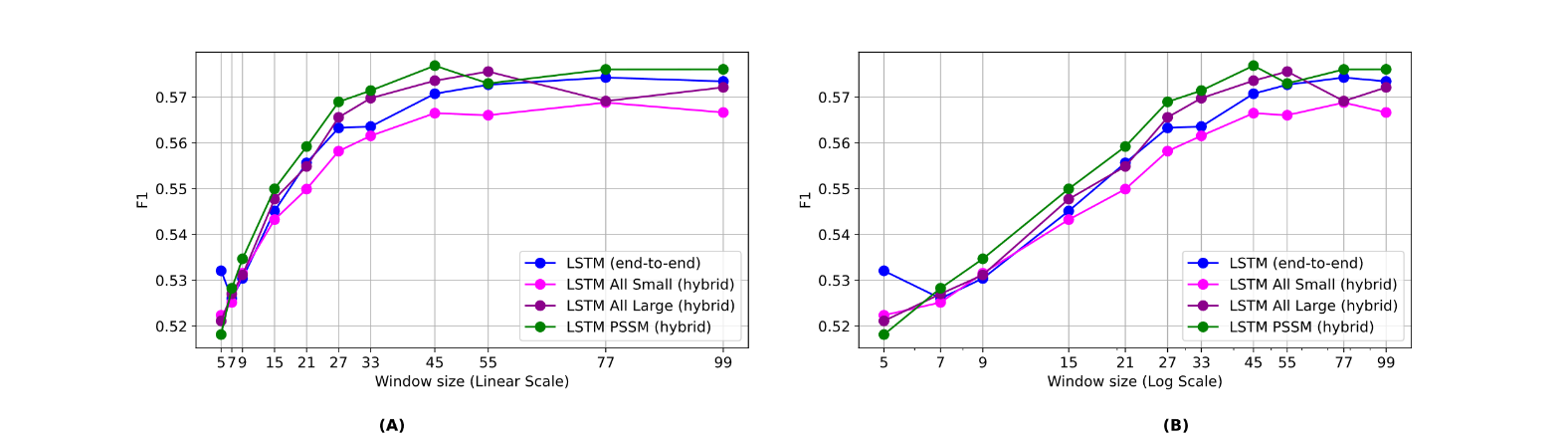


Figure S2: The difference between large and small LSTM models in Hybrid-based and sequence-based methods Macro-F1 score on the test set. **(A)** linear scale. **(B)** log scale.
